# Altered Serotonin 2A (5-HT_2A_) Receptor Signaling Underlies Mild TBI-Elicited Deficits in Social Dominance

**DOI:** 10.3389/fphar.2022.930346

**Published:** 2022-07-15

**Authors:** Sean M. Collins, Christopher J. O’Connell, Evan L. Reeder, Sophia V. Norman, Kainat Lungani, Poornima Gopalan, Gary A. Gudelsky, Matthew J. Robson

**Affiliations:** ^1^ Division of Pharmaceutical Sciences, University of Cincinnati James L. Winkle College of Pharmacy, Cincinnati, OH, United States; ^2^ Department of Biological Sciences, University of Cincinnati College of Arts and Sciences, Cincinnati, OH, United States; ^3^ Neuroscience Graduate Program, University of Cincinnati College of Medicine, Cincinnati, OH, United States

**Keywords:** traumatic brain injury, serotonin, behavior, social dominance, serotonin 2A receptor (5-HT_2A_)

## Abstract

Various forms of traumatic brain injury (TBI) are a leading cause of disability in the United States, with the generation of neuropsychiatric complications such as depression, anxiety, social dysfunction, and suicidality being common comorbidities. Serotonin (5-HT) signaling is linked to psychiatric disorders; however, the effects of neurotrauma on normal, homeostatic 5-HT signaling within the central nervous system (CNS) have not been well characterized. We hypothesize that TBI alters specific components of 5-HT signaling within the CNS and that the elucidation of specific TBI-induced alterations in 5-HT signaling may identify novel targets for pharmacotherapies that ameliorate the neuropsychiatric complications of TBI. Herein, we provide evidence that closed-head blast-induced mild TBI (mTBI) results in selective alterations in cortical 5-HT_2A_ receptor signaling. We find that mTBI increases *in vivo* cortical 5-HT_2A_ receptor sensitivity and *ex vivo* radioligand binding at time points corresponding with mTBI-induced deficits in social behavior. In contrast, *in vivo* characterizations of 5-HT_1A_ receptor function revealed no effect of mTBI. Notably, we find that repeated pharmacologic activation of 5-HT_2A_ receptors post-injury reverses deficits in social dominance resulting from mTBI. Cumulatively, these studies provide evidence that mTBI drives alterations in cortical 5-HT_2A_ receptor function and that selective targeting of TBI-elicited alterations in 5-HT_2A_ receptor signaling may represent a promising avenue for the development of pharmacotherapies for TBI-induced generation of neuropsychiatric disorders.

## Introduction

Traumatic brain injury (TBI) is a large-scale public health problem (Corso et al., 2015; Taylor et al., 2017). Although the etiology of TBI is heterogeneous in nature, various symptoms post-injury, including neuropsychiatric complications, are prevalent across various injury modalities (Ledreux et al., 2020). There are currently no FDA-approved pharmacotherapies to ameliorate either the acute effects of injury or enduring deficits that greatly effect the quality of life for those afflicted. Closed head mild TBI (i.e., concussion) is the most common clinical type of TBI, impacting over a million Americans annually, of which nearly 25% will have symptoms that persist for greater than 3 months post-injury (Ledreux et al., 2020). In addition, blast-induced TBI (bTBI) has been dubbed the “signature injury” of modern military conflicts (Agoston et al., 2017) and is known to result in the generation of enduring neuropsychiatric disorders (Mendez et al., 2013; Mac Donald et al., 2017a; Mac Donald et al., 2017b; Baker et al., 2018). Although decades of clinical and preclinical studies have greatly aided in our understanding of the symptoms and pathology associated with various forms of TBI, the precise molecular mechanisms driving the enduring effects of injury, and notably the psychiatric complications are still relatively unknown.

Alterations in serotonin (5-HT) signaling are linked to several neuropsychiatric disorders including major depressive disorder (MDD), anxiety, and PTSD (Davis et al., 1997; Frick et al., 2016) with more recent work linking 5-HT signaling within the central nervous system (CNS) to the complex regulation of social behavior (Dolen et al., 2013; Niederkofler et al., 2016; Walsh et al., 2018a; Robson et al., 2018). 5-HT neurons reside in the midbrain raphe nucleus and project to various brain regions including the cortex, hippocampus, and amygdala (Ren et al., 2018; Ren et al., 2019). 5-HT signaling within the CNS and periphery is tightly regulated by mechanisms that influence its synthesis, release, clearance by the presynaptic serotonin transporter (SERT), and signaling through endogenous receptors. The receptor subfamilies for 5-HT contain at least 14 members (Zmudzka et al., 2018), of which the 5-HT_1_ and 5-HT_2_ subfamilies are consistently linked to the emergence of neuropsychiatric disturbances and psychological disorders, including major depressive disorder (MDD) and altered social function (Carhart-Harris and Nutt, 2017; Walsh et al., 2018a; Zmudzka et al., 2018). Blast-induced TBI is reported to increase mRNA levels of tryptophan hydroxylase-2 (*tph-2*), the rate-limiting enzyme required for 5-HT production (Walther et al., 2003), in the dorsal raphe nucleus (DRN) (Kawa et al., 2015; Kawa et al., 2018). Controlled cortical impact (CCI) and penetrating injury disrupt 5-HT neuron tracks in the cerebral cortex, with significant regrowth occurring over time (Jin et al., 2016; Kajstura et al., 2018). These tracts, however, form altered spatial orientations (Jin et al., 2016; Kajstura et al., 2018), an effect with unknown implications on receptor expression/densities, neuronal network function, and behavior. Last, several studies have provided evidence that specifically targeting 5-HT_1A_ receptors post-injury may be advantageous in preventing cognitive/learning deficits (Kline et al., 2002; Olsen et al., 2012; Cheng et al., 2016), neurodegeneration (Kline et al., 2004; Cheng et al., 2016), and despair-like behavior post-injury in rodent subjects (Kosari-Nasab et al., 2019). It is currently clear that specific types of neurotrauma, including concussions, alter 5-HT signaling within the CNS (Jin et al., 2016; Kajstura et al., 2018); however, the extent to which 5-HT alterations impact psychiatric states, and ultimately behavior following neurotrauma, is currently not well characterized.

Of the regions within the CNS that are innervated by 5-HT circuitry, the prefrontal cortex has been implicated in higher-order tasks, including those that are dependent on the serotonergic activity like behavioral flexibility, comprehension of social hierarchies, and appropriate social function (Puig and Gulledge, 2011). Social deficits are intrinsically associated with prefrontal cortex function as exclusive, unilateral injury to the prefrontal lobe in mice was found to dramatically affect social recognition (Chou et al., 2016). Cases of unilateral injury to a particular brain region, however, are less common contrasted against the prevalence of injuries like concussions, which is the most common form of neurotrauma.

We hypothesized that various forms of mTBI may ultimately drive social dysfunction by disturbing the functional integrity of 5-HT synaptic circuitry within the prefrontal cortex specifically, as this region is implicated in social function dependent on 5-HT transmission. Elucidation of the underlying biologic adaptations occurring within the 5-HT architecture of the CNS in response to various forms of neurotrauma may provide more specific drug/therapeutic targets than currently utilized 5-HT reuptake inhibitors (SRI’s), which fail to produce desired pharmacologic effects in many individuals suffering from either MDD or neurotrauma-associated neuropsychiatric disorders (Kreitzer et al., 2019). The current study utilizes a murine model for closed-head blast-induced mTBI to characterize functional disruptions of *in vivo* 5-HT_2A_ receptor signaling within the CNS and to delineate whether identified alterations in 5-HT_2A_ receptor signaling are associated with aberrant social behavior elicited by neurotrauma.

## Materials and Methods

### Animals

All procedures were performed in compliance with the Institutional Animal Care and Use Committee (IACUC) at the University of Cincinnati. Adult, male wild-type C57Bl/6J (Jackson Labs Strain No: 000,664) subjects between the ages of 9–16 weeks were used for all experiments. Subjects were group-housed (≤4/cage) with food and water provided *ad libitum*, and maintained at the University of Cincinnati vivarium on a 14:10 light/dark cycle. All animals were housed within cages (Alternative Design Inc., Siloam Springs, AR) with spatial dimensions of 7.625 inches wide x 7.110 inches tall x 15.680 inches long (194 mm wide x 181 mm tall x 398 mm long) to allow for unrestricted movement on a floor space of 77.5 in^2^ | 500 cm^2^. Cages are routinely cleaned by University of Cincinnati vivarium staff once every two weeks or as needed and are monitored continuously to ensure a sanitary home-cage environment. All animals were housed within a temperature- and humidity-regulated room. Cage temperature is passively regulated by the 74°F set point established by the temperature within the husbandry facility. The humidity in the husbandry facility fluctuates between 30% and 70% saturation. All experimental procedures were conducted during the light cycle following a one-week facility acclimation. All animals within experimental groups were housed in the same rooms within the husbandry facility.

### Blast-Induced Closed Head Mild Traumatic Brain Injury

Blast-induced mild TBI (mTBI) was induced as previously described (Logsdon et al., 2017; Logsdon et al., 2020). In brief, following a 30-min acclimation period, sham and mTBI subjects were anesthetized with 4% volume-to-volume isoflurane (VetEquip, Plane III, paralysis of intercostal muscles). Subjects were positioned into a padded polyvinylchloride (PVC) shielding apparatus, perpendicular to the blast wave front, shielding internal organs from injury. Consistency in alignment was maintained using each respective subject’s anatomy, aligning the occipital condyle with the precise edge of protective shielding, allowing consistent head acceleration/deceleration per subject. The blast device consisted of a 2-piece machined steel shock tube apparatus driven using compressed helium gas [23]. Sham subjects were exposed to anesthesia, a shielding tube, and noise without exposure to the shock wave. Following mTBI or sham treatments, subjects were immediately removed, and time to regain righting reflex (RRT) was measured prior to subjects being returned to their respective home cages. Experimental groups consisted of mTBI or sham subjects, subsequently randomized to pharmacologic treatments, behavioral paradigms, and/or molecular characterizations.

### 5-HTP- and DOI-Induced Head Twitch Response

5-Hydroxy-l-tryptophan (5-HTP)-induced head twitch assays were conducted as previously described (Schmid et al., 2008). Subjects were administered 5-HTP (100 mg/kg) or vehicle, and 30 min post-administration, head twitch response (HTR) was monitored for 10 min by two independent blinded reviewers. 2,5-Dimethoxy-4-iodoamphetamine (DOI, 1 mg/kg i.p., Sigma Aldrich, St. Louis, MO)-induced HTR assays were conducted as previously described, with HTR being monitored for 10 min by two independent blinded reviewers (Canal et al., 2010; Veenstra-VanderWeele et al., 2012). In brief, HTR was visually quantified by trained, blinded reviewers 34 min following administration of DOI (1mg/kg) or vehicle treatments. All compounds were administered *via* intraperitoneal injection.

### 8-OH-DPAT-Induced Hypothermia Assay

5-HT_1A_ receptor sensitivity was assessed as previously described (Veenstra-VanderWeele et al., 2012). Ten days following mTBI or sham treatments, subjects were acclimated to the testing facility for at least 30 min and the 5-HT_1A_ receptor agonist 8-OH-DPAT (0.1 mg/kg, s. c., Sigma Aldrich, St. Louis, MO) was administered. Subjects were returned to their home cages and core body temperature was recorded every 10 min for 60 min in a blinded fashion.

### Quantitative Real-Time PCR

To interrogate changes in 5-HT_2_a mRNA expression, total RNA was extracted from flash-frozen bilateral prefrontal and somatosensory cortices (*N* = 4–5/experimental group) using TRI Reagent (Sigma-Aldrich, T9424) in accordance with the manufacturer’s instructions and subjected to TaqMan^®^ quantitative real-time PCR (qRT-PCR). Total RNA concentrations for each sample were quantified using a NanoDrop™ One (Thermo Scientific, Waltham, MA). Samples of cDNA were generated by reverse transcription reaction using a high-capacity reverse transcription kit (Applied Biosystems, Foster City, CA) using 100 µg total RNA/sample. Each sample reaction included MultiScribe TM Reverse Transcriptase and random primers, and was run per the manufacturer’s guidance at the following thermal cycler conditions: step 1 at 25°C for 10 min, step 2 at 37°C for 120 min, step 3 at 85°C for 5 s, and step 4 at 4°C for 10 min.

In conducting PCR amplification, TaqMan^®^ Universal PCR Master Mix and the following probes were obtained from Applied Biosystems (Foster City, CA): *18* *s* (Hs99999901_s1) as an endogenous control gene and *htr2a* (*Mm00555764_m1*). The reaction mixture was prepared according to the manufacturer’s instructions, with the following thermal cycling conditions: initial holding at 50°C for 2 min, followed by a first denaturing step at 95°C for 10 min, then 40 cycles at 95°C for 15 s, and at 60°C for 1 min. Data obtained from qRT-PCR measurements were calculated using the ΔΔC_t_ method.

### [^3^H]Ketanserin Binding Assay

5-HT_2A_ binding was analyzed in membrane preparations as previously described (Canal et al., 2010). Three or 10 days following mTBI or sham treatments, subjects were sacrificed *via* rapid decapitation and bilateral frontal cortex samples were dissected on ice. Samples were homogenized in 3 ml ice-cold Tris binding assay buffer (50 mM Tris-HCl, 10 mM MgCl_2_, 0.1 mM EDTA, pH = 7.4), and subsequently centrifuged at 20,000 x g for 20 min at 4°C. The supernatant was decanted, and the pellet was resuspended in a 1.5-ml binding assay buffer. Protein concentrations were measured with a Pierce BCA Protein Assay Kit (Thermo Fisher Scientific, Waltham, MA). Membrane preparations (200 µg protein) were incubated with the 5-HT_2A_ antagonist [^3^H]ketanserin (PerkinElmer, Waltham, MA) (1 or 10 nM) at 37°C for 60 min. Samples were run in duplicate and nonspecific binding was determined in the presence of 100 µM methysergide (Sigma Aldrich, St Louis, MO). Samples were collected using a Brandel cell harvester and washed with ice-cold phosphate-buffered saline (PBS, pH = 7.4). Samples were incubated in 7 ml scintillation fluid overnight (National Diagnostics) and radioactive counts were measured *via* scintillation spectrometry (Beckman Coulter).

### Locomotor Activity Analysis

To delineate acute functional effects of mTBI, three hours post-injury or sham treatments, locomotor activity was assessed using clear acrylic chambers (28 cm × 28 cm) in combination with video tracking software (ANY-Maze, Stoelting Co., Wood Dale, IL). Subjects were monitored for 30 min and total locomotor activity was quantified.

### Crawley Three-Chamber Test for Sociability

Ten days following mTBI, sociability was assessed utilizing the Crawley Three Chamber Sociability Assay (Yang et al., 2011). One day prior to testing, mice were acclimated to an empty acrylic three-chamber apparatus for 10 min. Time spent in each chamber was recorded using video tracking software (ANY-maze). Any subject with a chamber preference, as assessed during the acclimation period, was excluded from further study. The social chamber contained a wire pencil cup housing a novel age- and sex-matched mouse, previously habituated to the pencil cup, and the object chamber contained an empty pencil cup. Time spent in each chamber as well as total distance traveled was recorded, and differences between groups were analyzed. A zone immediately surrounding the wire pencil cup housing the novel mouse was identified as a social interaction zone. A separate identical zone surrounded the empty pencil cup on the opposing side of the acrylic three-chamber apparatus. Time spent in the zones directly surrounding the pencil cups was quantified and analyzed, and investigation of each chamber and deliberate exploration of either wire pencil cup was quantified and analyzed.

### Tube Test for Social Dominance

Changes in social aggression and/or dominance were analyzed using the tube test for social dominance 10 days following mTBI (Veenstra-VanderWeele et al., 2012). mTBI and sham subjects were acclimated to a 14” acrylic tube for two days prior to testing. Subjects that did not pass through the tube were excluded from further analysis. For any given pairing, dominance was determined as full traversal through the tube requiring the non-dominant subject to back out of the tube. The number of dominant trials for each group was recorded and analyzed by experimenters blinded to the treatment group. For the DOI reversal experiment, DOI administration (0.1 mg/kg, i.p., QD x 8 days) was initiated three days post-injury, at a time point where we experimentally identified mTBI-induced alterations in 5-HT_2A_ receptor sensitivity.

### Statistical Analyses

All statistical analyses were conducted in GraphPad Prism 8. Sample sizes for all experiments have been estimated using an *α* = 0.05 and power = 80%, along with expected means and standard deviation from our own generated preliminary data and/or relevant previously published studies. One- or two-way ANOVAs preceded *post hoc* Bonferroni's or Sidak’s multiple comparison analyses where applicable. Locomotor and hypothermia assays were analyzed using two-way repeated-measures ANOVA followed by *post hoc* Bonferroni's multiple comparison analyses. Unpaired t-tests were used where applicable. Binomial data obtained using the tube test for social dominance were analyzed using Fisher’s exact tests. For all statistical analyses, *p* ≤ 0.05 was considered statistically significant.

## Results

### Blast-Induced Mild Traumatic Brain Injury Increases Righting Reflex Time and Decreases Acute Locomotor Activity

**FIGURE 1:**
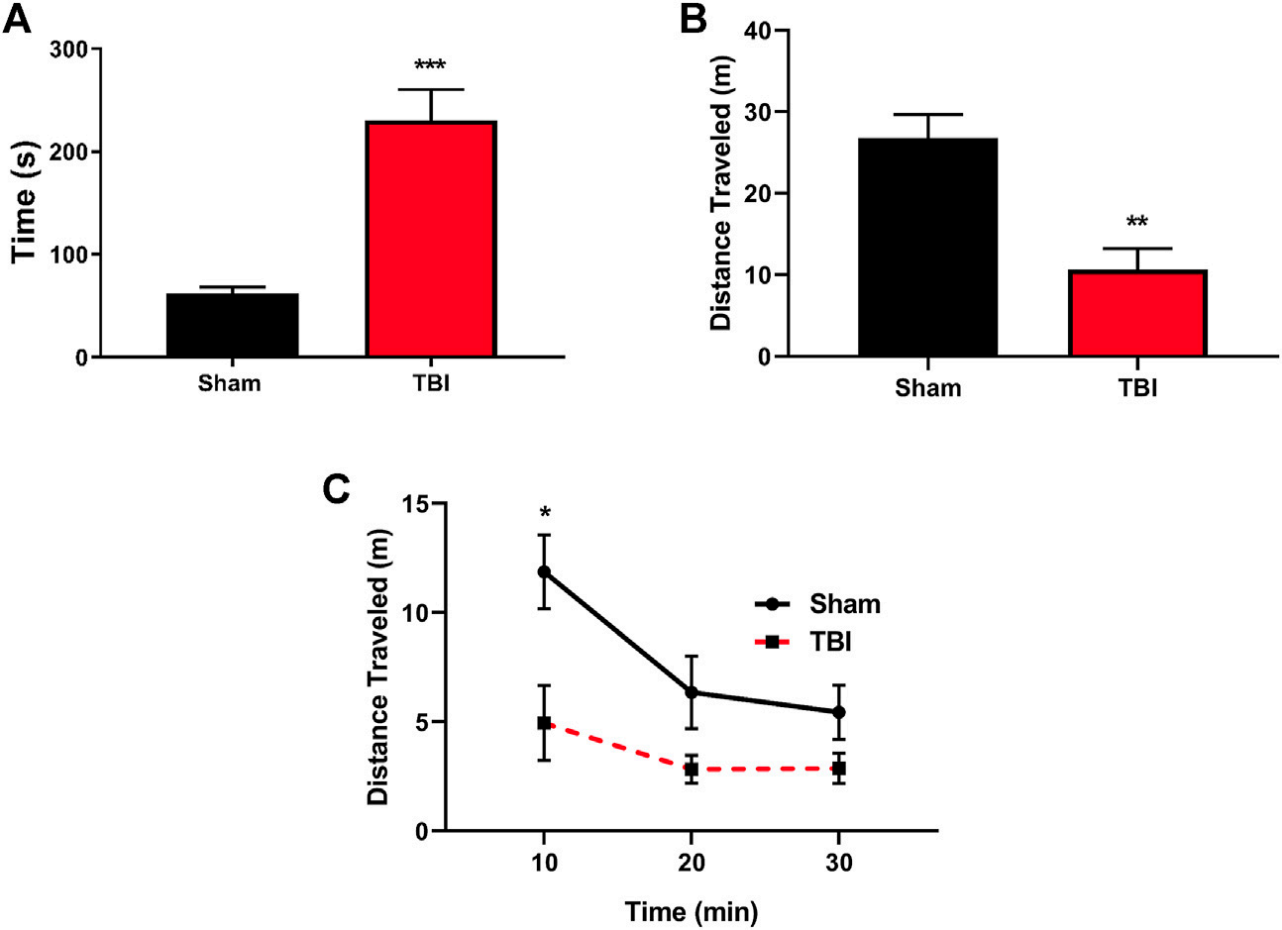
mTBI results in significant increases in RRT and altered locomotion following the injury. **(A)** mTBI results in significant increases in righting reflex time (RRT), indicative of a loss of consciousness in mTBI subjects as compared to their sham counterparts immediately following injury paradigm (*t* = 5.31, ***p ≤ 0.0001, *N* = 11 sham, 12 TBI). **(B)** Total locomotor activity three hours post-mTBI or sham paradigm. mTBI subjects exhibit a significant reduction in total locomotor activity acutely following injury paradigm (*t* = 4.22, **p ≤ 0.001, *N* = 7/group). **(C)** Locomotor activity binned over time revealed that mTBI subjects exhibit a reduction in exploratory activity immediately upon exposure to a novel environment [F (1,12) = 7.644, *p* = 0.017, *t* = 2.870, *p ≤ 0.05, *N* = 7/group] as compared to their sham counterparts.

One of the acute effects of TBI includes loss of consciousness, and herein, we utilized righting reflex time (RRT) as a murine correlate of injury-elicited unconsciousness (Creed et al., 2011; Dams-O'Connor et al., 2013). Subjects exposed to mTBI paradigm exhibited significant increases in RRT compared to their sham counterparts (Figure 1A). Second, locomotor analysis three hours post-injury (Figure 1B) revealed a significant injury-induced decrease in general locomotor activity in TBI-exposed subjects and their sham counterparts (*t = 4.22, p* ≤ 0.001). Locomotor activity differences stem from exploratory locomotor activity upon entrance into a novel environment (Figure 1C) with a significant difference in locomotor activity seen during the initial 10-min exploratory period (*t* = 2.870, *p* ≤ 0.05).

### Characterization of 5-HT Receptor Hypersensitivity Following Blast-Induced Mild Traumatic Brain Injury

**FIGURE 2:**
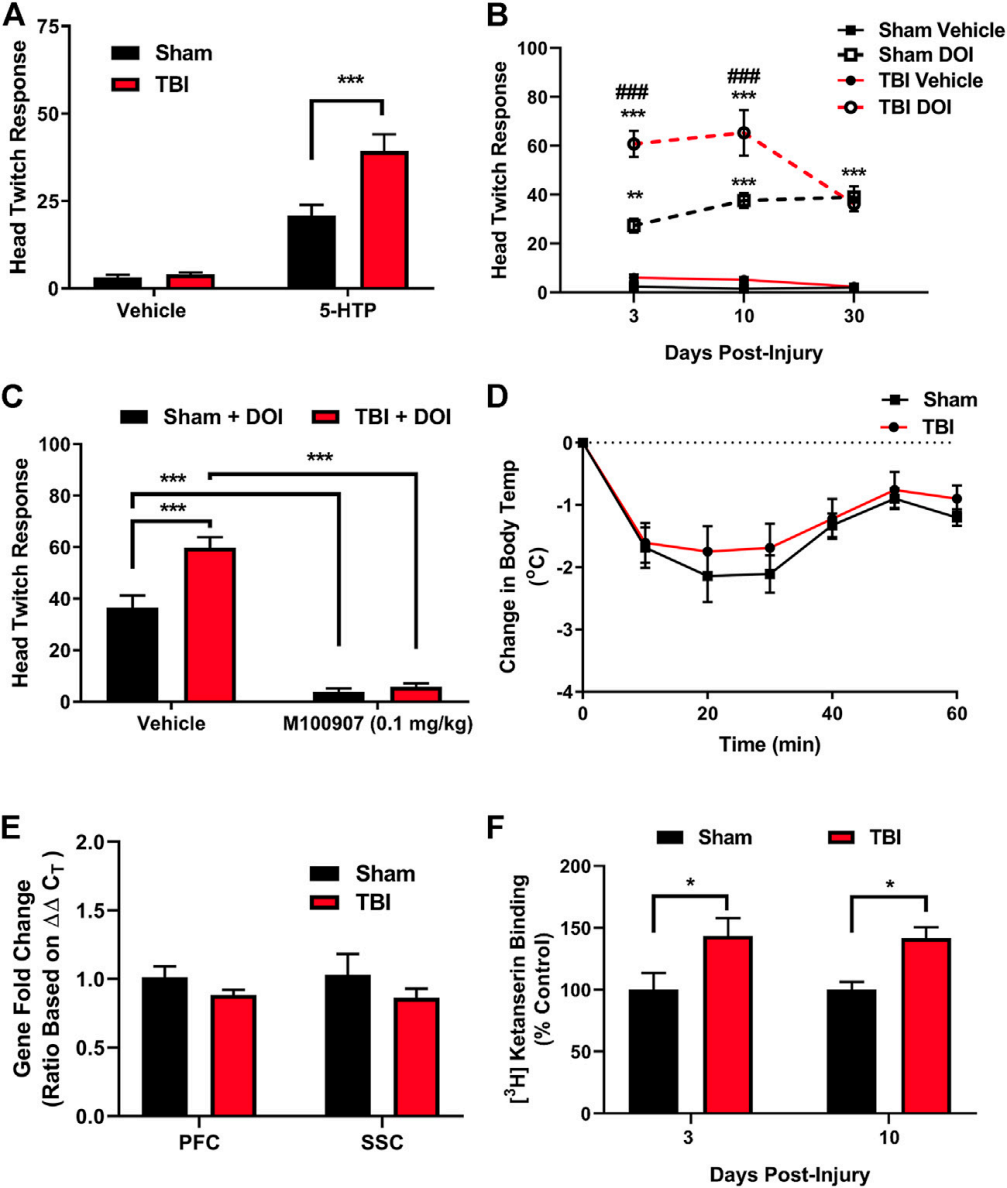
mTBI results in 5-HT_2A_ receptor hypersensitivity and increased cortical 5-HT_2A_ receptor binding. **(A)**. 5-Hydroxytryptophan (5-HTP) administration (100 mg/kg, i.p.) results in a prototypic head twitch response (HTR), an effect derived from cortical 5-HT_2A_ receptor activation. mTBI subjects exhibit a significant potentiation in HTR as compared to their sham counterpart subjects ten days post-injury [F (1,40) = 11.39, *p* = 0.0017, *N* = 11/group, *post hoc* analysis *t* = 4.57, ***p ≤ 0.001]. **(B)** Administration of the selective 5-HT_2A_ receptor agonist DOI [1.0 mg/kg, i.p.] results in significant increases in HTR as compared to vehicle in sham subjects [F (3,85) = 118.1, *p* ≤ 0.0001, *N* = 6–10/group, *post hoc* analysis 3 days post-sham (*N* = 8 Sham-Vehicle, 10 TBI-Vehicle, 8 Sham-DOI, 9 TBI-DOI, *t* = 4.47, **p = 0.001), 10 days post-sham (*N* = 9 Sham-Vehicle, 10 TBI-Vehicle, 8 Sham-DOI, 9 TBI-DOI, t = 6.63, ***p ≤ 0.0001], and 30 days post-sham (*N* = 7 Sham-Vehicle, 6 TBI-Vehicle, 7 Sham-DOI, 6 TBI-DOI, t = 6.20, ***p ≤ 0.0001). Administration of DOI in mTBI subjects results in a potentiation of DOI-induced HTR, indicative of an increase in cortical 5-HT_2A_ receptor sensitivity, as compared to sham subjects [F (6,85) = 4.39, *p* ≤ 0.001] both 3 (t = 6.14, ###*p* ≤ 0.0001) and 10 (t = 5.10, ###*p* ≤ 0.0001) days post-injury, an effect absent by 30 days post-injury. **(C)** Pretreatment with the 5-HT_2A_ receptor antagonist M100907 mitigates DOI-induced HTR in sham subjects [Treatment (F (1,25) = 188.2, *p* ≤ 0.0001]; Interaction [F (1,25) = 11.37, *p* ≤ 0.01, *N* = 6 Sham-Vehicle-DOI, 8 TBI-Vehicle-DOI, 8 Sham-M100907-DOI, 7 TBI-M100907-DOI, *t* = 7.17, ***p ≤ 0.0001] and the potentiation of DOI-induced HTR in TBI subjects (t = 12.35, ***p ≤ 0.0001), indicative of a dependence on intact 5-HT_2A_ receptor signaling in the mTBI-induced potentiation of DOI HTR. **(D)** Administration of the 5-HT_1A_ agonist 8-OH-DPAT (0.1 mg/kg, i.p.) results in a significant, transient reduction in core body temperature in sham subjects, an effect unaltered by mTBI ten days post-injury (*N* = 12 Sham, 10 TBI), providing *in vivo* evidence of selective effects of mTBI on 5-HT_2A_ receptor sensitivity. **(E)** No differences were found in *htr2A* (5-HT_2A_ receptor) mRNA expression in mTBI subjects in either the PFC or SSC, as compared to their sham counterparts ten days post-injury (N = 5/group). **(F)** *Ex vivo* 5-HT_2A_ receptor binding assays revealed mTBI-induced increases in 5-HT_2A_ receptor binding within the frontal cortex at both 3 (*N* = 8/group, *t* = 2.18, *p ≤ 0.05) and 10 (*N* = 6 Sham, 7 TBI, *t* = 3.80, *p ≤ 0.05) days post-injury, time points corresponding to mTBI-induced potentiation of 5-HT_2A_-mediated HTR.

5-HTP is rapidly converted to 5-HT *in vivo* and acts to increase synaptic levels of 5-HT, affording the opportunity to ubiquitously probe 5-HT receptor signaling sensitivity through pharmacologic means (Schmid et al., 2008; Schmid and Bohn, 2010). 5-HTP administration (100 mg/kg, i.p.) through intraperitoneal injection in subjects ten days post-injury or sham procedures revealed a significant potentiation of HTR (Figure 2A), an effect mediated by cortical 5-HT_2A_ receptors that requires *β*-arrestin signaling (Schmid et al., 2008). DOI is a selective 5-HT_2A_ receptor agonist that results in HTR in a *β*-arrestin-independent manner (Schmid et al., 2008). The administration of DOI to sham and mTBI subjects resulted in the mTBI-induced potentiation of DOI-elicited HTR (Figure 2B), an effect present 3 days post-injury (*t* = 4.88, *p* ≤ 0.0001) and 10 days post-injury (*t* = 4.95, *p* ≤ 0.0001), before returning to control levels 30 days post-injury (*t* = 0.44, n. s.), providing further evidence of increases in cortical 5-HT_2A_ receptor modulation by mTBI. It should be noted that we have conducted similar preliminary studies in female subjects 10 days post-injury or sham treatments, and found a corresponding mTBI-elicited increase in DOI-induced HTR (data not shown). As DOI-induced HTR can be altered by several receptors, including mGluR2, 5-HT_2C_, and sigma receptors (Gewirtz and Marek, 2000; Malik et al., 2016), we pharmacologically characterized whether intact 5-HT_2A_ receptor activity is required for the TBI-elicited potentiation of DOI-induced HTR using the 5-HT_2A_ receptor antagonist M100907 (0.1 mg/kg, i.p.). M100907 administration was found to attenuate both DOI-induced HTR in sham subjects (Figure 2C) and mTBI-elicited potentiation of DOI-induced HTR (*t* = 12.35, *p* ≤ 0.0001), indicating a requirement of intact 5-HT_2A_-mediated signaling in mTBI-induced hypersensitivity to DOI administration. We next sought to ascertain if observed mTBI-induced 5-HT_2A_ hypersensitivity extends to other 5-HT receptor subtypes such as 5-HT_1A_ receptors. Using *in vivo* 8-OH-DPAT hypothermia assays as a determination of 5-HT_1A_ receptor sensitivity, we found no discernable difference in 5-HT_1A_ receptor sensitivity 10 days post-injury (Figure 2D), indicating some level of specificity in mTBI-elicited alterations of *in vivo* 5-HT receptor sensitivity.

### Blast-Induced Mild Traumatic Brain Injury Increases in Cortical 5-HT_2A_ Receptor Binding

We hypothesized that observed *in vivo* increases in 5-HT_2A_ receptor sensitivity elicited by mTBI may stem from increases in the cortical expression of 5-HT_2A_ receptors. TaqMan quantitative real-time PCR (qRT-PCR) assessment of *htr2a* (5-HT_2A_) mRNA expression in prefrontal cortex (PFC) or somatosensory cortex (SSC) samples from either mTBI or sham subjects revealed no alterations in overt expression at 10 days post-injury (Figure 2E). We next sought to delineate whether mTBI acts to increase ligand binding to cortical 5-HT_2A_ receptors. Protein level analysis using [^3^H]ketanserin radioligand binding assays in frontal cortex samples obtained from sham or mTBI subjects revealed increased 5-HT_2A_ receptor binding (Figure 2F) at time points 3 (*t* = 2.18, *p ≤ 0.05) and 10 (*t* = 3.80, *p ≤ 0.05) days post-injury correlating with observed mTBI-elicited 5-HT_2A_ hypersensitivity.

### Abnormalities in Sociability and Social Dominance Following Mild Traumatic Brain Injury Are Time-dependent

**FIGURE 3:**
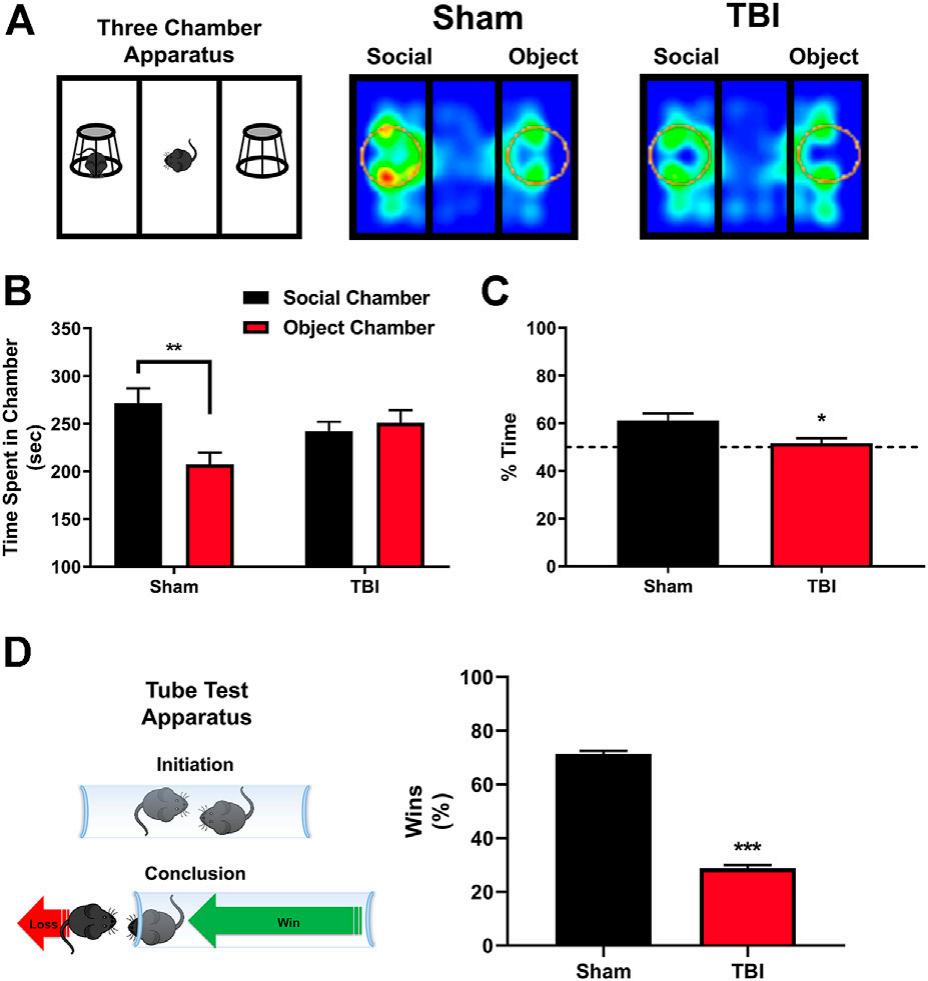
mTBI results in deficits in sociability and altered social dominance. **(A)** Depiction of Crawley three-chamber sociability apparatus and representative heatmaps of sham and mTBI subjects ten days post-injury. mTBI subjects exhibit a reduction in interaction time with a novel conspecific, indicative of a reduction in general sociability. **(B)** Quantification of total time spent in the respective social chamber as compared to object chamber by sham or mTBI subjects. Sham mice exhibit a clear, significant preference for time spent in the social chamber containing a novel, gender, and age-matched conspecific subject [F (1,32) = 7.47, *p* ≤ 0.01, N = 10 Sham, 8 TBI, *post hoc* analysis (t = 3.57, **p ≤ 0.01], in stark contrast to mTBI subjects which fail to elicit any preference for the social interaction chamber as compared to the object chamber (*t* = 0.45, n. s.) 10 days post-injury. **(C)** Quantification of percent time spent specifically within the social interaction zone (depicted as circles within apparatus in 3A) with the novel, conspecific by sham and mTBI subjects 10 days post-injury. mTBI subjects fail to exhibit a significant social interaction zone preference, whereas sham subjects exhibit a clear social preference (*t* = 2.53, *p ≤ 0.05). **(D)** In concordance with deficits in sociability, the tube test for social dominance (depiction included) revealed a significant decrease in social dominance behavior 10 days post-injury in mTBI subjects (Fisher’s exact test, ***p ≤ 0.001, *N* = 15/group) when compared to their sham counterparts.

Alterations in 5-HT_2A_ receptor expression on oxytocin-containing neurons are linked to altered social behavior (Edwards et al., 2018), and targeting 5-HT_2A_ receptors has been shown to reverse aberrant behaviors in murine models for autism spectrum disorder (ASD) (Amodeo et al., 2017; Panzini et al., 2017). Social behavior changes are prevalent following TBI (Milders, 2018), with aggression and/or social apathy being present in approximately 35% of those afflicted (Schwarzbold et al., 2008), an effect recapitulated in preclinical neurotrauma rodent models (Semple et al., 2018). Using the Crawley Three-Chamber Sociability assay 10 days post-injury or sham procedures, we find that sham subjects demonstrate an as-expected social preference, measured by increases in time spent in the social chamber compared with the object chamber (Figures 3A,B), and an increased social preference index (Figure 3C, *t* = 2.53, *p ≤ 0.05), an effect not observed in TBI subjects. Similarly, in Figure 3D, using the tube test for social dominance as depicted, we find significant reductions in the social dominance behavior exhibited by mTBI subjects ten days post-injury as compared to their sham counterparts.

### Low-Dose Repeated Administration of 5-HT_2A_ Receptor Agonist DOI Normalizes Mild Traumatic Brain Injury-Elicited Deficits in Social Dominance

**FIGURE 4:**
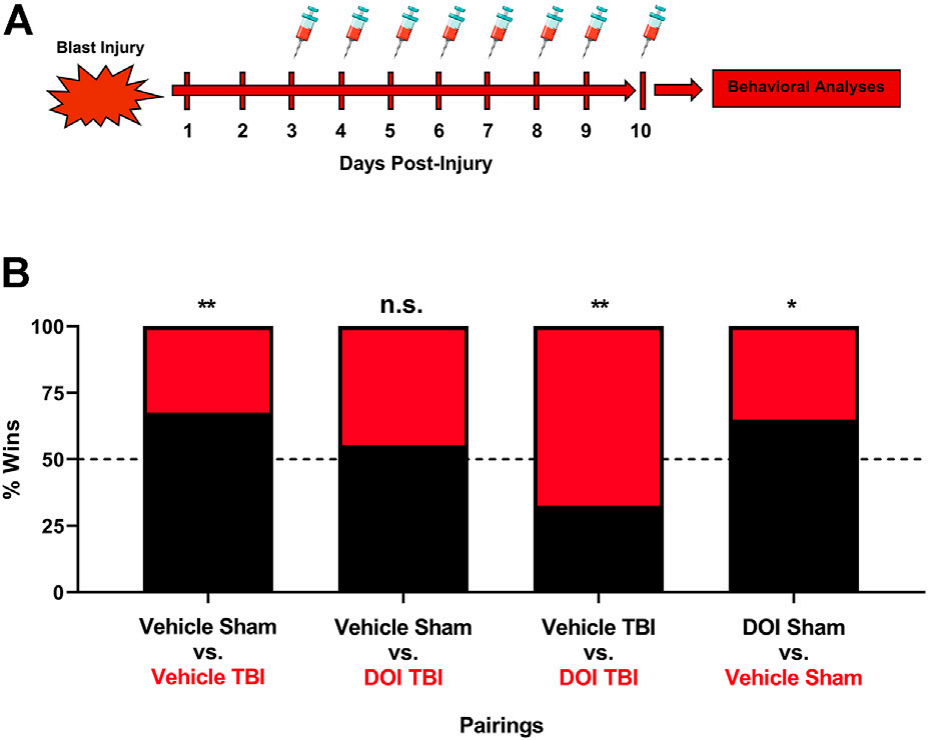
Repeated pharmacologic 5-HT_2A_ receptor activation ameliorates mTBI-elicited deficits in social dominance. **(A)** Low-dose DOI administration (0.1 mg/kg, **(I)** p., QD) was initiated three days post-injury or sham procedures, a time point where increased 5-HT_2A_ receptor sensitivity is noted following mTBI. **(B)** One hour post-DOI administration 10 days post-injury, subjects were paired off in the tube test for social dominance. Again, we found that mTBI subjects administered vehicle exhibited a reduction in social dominance 10 days post-injury as compared to their sham counterparts (vehicle sham vs. vehicle mTBI, Fisher’s Exact Test, **p ≤ 0.01, *N* = 12/group). DOI administration in mTBI subjects attenuated social dominance deficits when compared to their sham vehicle administered counterparts (vehicle sham vs DOI mTBI, Fisher’s exact test, n. s., *N* = 12/group). Furthermore, mTBI subjects administered DOI exhibit a significant increase in social dominance behavior as compared to their vehicle-treated mTBI counterparts (vehicle mTBI vs. DOI mTBI, Fisher’s exact test, **p ≤ 0.01, *N* = 12/group). It should be noted that DOI administration in sham subjects exerts a small, albeit significant increase in social dominance behavior as compared to vehicle-treated sham subjects (vehicle sham vs. DOI sham, Fisher’s exact test, *p ≤ 0.05, N = 12/group), an effect pointing to a role of 5-HT_2A_ receptor signaling in regulating the social dominance behavior under normal physiologic conditions.

To delineate if altered cortical 5-HT_2A_ receptor function elicited by mTBI is involved in disrupted social dominance, we repetitively administered low-dose DOI (0.1 mg/kg x 8 days) or vehicle to sham or mTBI subjects. Importantly, dosing of vehicle or DOI was initiated 3 days post-injury, a time point where we find significant increases in 5-HT_2A_ sensitivity in HTR assays (Figure 2). Again, as demonstrated in Figure 4, mTBI resulted in a reduction in social dominance 10 days post-injury. Notably, low-dose DOI administration resulted in a normalization of social dominance behaviors as mTBI subjects administered DOI exhibited no differences in social dominance as compared to vehicle-treated sham subjects. Additionally, repetitive administration of DOI in mTBI subjects reverses social dominance as compared to vehicle-treated mTBI subjects. We did find a smaller, albeit significant effect of repeated DOI administration on the social dominance behavior alone in line with the known role of 5-HT signaling in regulating complex social behaviors (Dolen et al., 2013; Niederkofler et al., 2016; Walsh et al., 2018a; Robson et al., 2018).

## Discussion

Herein, we provide evidence that closed-head blast-elicited mTBI results in defined alterations of cortical 5-HT_2A_ receptor sensitivity and ligand binding occurring with concomitant alterations in sociability and social dominance. Furthermore, data herein provide evidence that selective targeting of mTBI-elicited 5-HT_2A_ signaling alterations acts in reversing neurotrauma-induced deficits in social dominance. 5-HT signaling within the CNS is foundational in establishing social paradigms, as homeostatic 5-HT transmission sustains a variety of psychosocial phenomena, including normative social functioning (Dolen et al., 2013; Walsh et al., 2018a; Robson et al., 2018; Heifets et al., 2019). Among 5-HT receptor subtypes, specific targeting of the 5-HT_2A_ receptor has revealed that 5-HT_2A_ receptors are involved in observable features of social behavior across various models, including genetic models characterized by deficits in social behavior such as murine models for ASD (Guo and Commons, 2017; Panzini et al., 2017). The ability of serotonin signaling dependent on the 5-HT_2A_ receptor to modulate social function and mood across both injury- and genetic-derived models of altered social behavior underscores the capacity and robustness of the 5-HT_2A_ receptor as a target for investigation, and suggests that pharmacological interrogation of dysregulated 5-HT_2A_ signaling may be beneficial in the context of mTBI. Although we find that mTBI drives hypersensitivity of cortical 5-HT_2A_ receptors, we find that repetitive agonist-induced activation of 5-HT_2A_ receptors results in the normalization of aberrant social behaviors in murine subjects. Counterintuitively, the administration of 5-HT_2A_ receptor agonists results in the pharmacological process known as tachyphylaxis, which is the acute, rapid desensitization of receptors upon repetitive stimulation (Abbas et al., 2009; Buchborn et al., 2018). This includes decreases in detectable 5-HT_2A_ receptor densities and/or expression in cortical regions known to be involved in the actions of 5-HT_2A_ receptor agonists (Raval et al., 2021). We hypothesize that repetitive stimulation of mTBI-induced hypersensitive 5-HT_2A_ receptors results in the reversal of these effects, that is, the normalization of 5-HT_2A_ receptor sensitivity in mTBI subjects. Future studies aimed at the further characterization of mechanisms in which mTBI results in cortical 5-HT_2A_ receptor hypersensitivity and how agonist stimulation normalized aberrant social behaviors are clearly needed.

These findings expand upon a growing body of evidence supporting the therapeutic efficacy of 5-HT receptor agonists for the treatment of neuropsychiatric disturbances, and our data concur with a limited number of systematic studies aimed at determining the effects of various forms of TBI on central 5-HT signaling. As noted earlier, several previous studies have provided evidence that targeting 5-HT_1A_ receptors post-injury is advantageous in preventing cognitive/learning deficits (Kline et al., 2002; Olsen et al., 2012; Cheng et al., 2016), neurodegeneration (Kline et al., 2004; Cheng et al., 2016), and despair-like behavior post-injury in rodent subjects (Kosari-Nasab et al., 2019). Although we failed to find *in vivo* evidence of altered 5-HT_1A_ function post-injury, a singular study has shown increased hippocampal 5-HT_1A_ receptor expression post-injury (Wilson and Hamm, 2002). Recent studies have indicated that 5-HT_7_ receptors may be involved in the beneficial actions of 5-HT_1A_ receptor agonists (Yelleswarapu et al., 2012), providing evidence that these receptor interactions are inherently complicated and may be context-specific. The results from those studies are aligned with the data here, where we failed to detect a specific, functional difference in 5-HT_1A_ receptor sensitivity following closed head injury; however, it should be noted that we did not specifically ascertain whether 5-HT_7_ receptors are altered by mTBI. The functional roles of the various 5-HT receptor subtypes in the generation of social deficits and neuropsychiatric disturbances after mTBI are obfuscated by a high degree of system complexity, as 5-HT receptor subtypes exhibit both excitatory and inhibitor G-protein-coupled signaling pathways, biased agonism, and various protein–protein interactions, and it is possible that mTBI-induced alterations in 5-HT signaling may range from the receptor, and synaptic and/or neurocircuitry level.

It should be noted that clinical studies have established that SRI administration results in minimal efficacy for neuropsychiatric complications stemming from various forms of neurotrauma (Kreitzer et al., 2019). This is perhaps unsurprising, as these drugs have abysmal clinical success in treating non-trauma elicited major depressive disorder and further act to indiscriminately increase synaptic levels of 5-HT by blocking the synaptic clearance of 5-HT, thereby increasing 5-HT receptor activation in an unbiased manner (Alex and Pehek, 2007; Jakobsen et al., 2017). As a result of these clinical data and data contained herein, we propose that future, further delineation of specific neurotrauma-elicited alterations in various 5-HT receptor subtypes will provide viable, drug development opportunities beyond that of typically utilized SRIs.

Collectively, our studies provide crucial insight into the functional consequences of mTBI-induced alterations in 5-HT receptor signaling and reveal pharmacologically targetable alterations in 5-HT_2A_ receptor function that afford reversal of mTBI-elicited alterations in social function. There are caveats to our studies, including the wide age range and a lack of female subjects utilized for many of the current studies. A better understanding of the age, sex, and time dependence and/or regional specificities of alterations in the expression and/or function of various 5-HT receptors and/or 5-HT neurons following various forms of neurotrauma is certainly warranted. Studies herein aimed at exploring the nuances of 5-HT-mediated signaling following mTBI and may provide the groundwork for novel and effective clinical therapies to improve patient outcomes following TBI.

## Conclusion

mTBI acts to potentiate cortical 5-HT_2A_ receptor sensitivity and increase ligand binding, effects independent of simple transcriptional upregulation of the receptor. mTBI-induced increases in 5-HT_2A_ receptor sensitivity occur at the time points of altered social behavior and repeated administration of a selective 5-HT_2A_ receptor agonist was found to normalize social dominance deficits stemming from mTBI. These studies provide a potential neurobiological basis and rationale for the further study of drugs targeting 5-HT_2A_ receptors in treating TBI-induced neuropsychiatric complications.

## Data Availability

The raw data supporting the conclusions of this article will be made available by the authors, without undue reservation.
